# Impact of Oil on Bacterial Community Structure in Bioturbated Sediments

**DOI:** 10.1371/journal.pone.0065347

**Published:** 2013-06-10

**Authors:** Magalie Stauffert, Cristiana Cravo-Laureau, Ronan Jézéquel, Sandra Barantal, Philippe Cuny, Franck Gilbert, Christine Cagnon, Cécile Militon, David Amouroux, Fatima Mahdaoui, Brice Bouyssiere, Georges Stora, François-Xavier Merlin, Robert Duran

**Affiliations:** 1 Equipe Environnement et Microbiologie, Institut Pluridisciplinaire de Recherche en Environnement et Matériaux, Université de Pau et des Pays de l’Adour, Pau, France; 2 Centre de Documentation, de Recherche et d'Expérimentations sur les pollutions accidentelles des Eaux, Brest, France; 3 Equipe Microbiologie Environnement et Biotechnologie, Mediterranean Institute of Oceanography, Aix-Marseille Université, Marseille, France; 4 Laboratoire Ecologie Fonctionnelle et Environnement, Université de Toulouse, Toulouse, France; 5 Equipe Chimie Analytique Bio-Inorganique et Environnement, Institut Pluridisciplinaire de Recherche en Environnement et Matériaux, Université de Pau et des Pays de l’Adour, Pau, France; Argonne National Laboratory, United States of America

## Abstract

Oil spills threaten coastlines where biological processes supply essential ecosystem services. Therefore, it is crucial to understand how oil influences the microbial communities in sediments that play key roles in ecosystem functioning. Ecosystems such as sediments are characterized by intensive bioturbation due to burrowing macrofauna that may modify the microbial metabolisms. It is thus essential to consider the bioturbation when determining the impact of oil on microbial communities. In this study, an experimental laboratory device maintaining pristine collected mudflat sediments in microcosms closer to true environmental conditions – with tidal cycles and natural seawater – was used to simulate an oil spill under bioturbation conditions. Different conditions were applied to the microcosms including an addition of: standardized oil (Blend Arabian Light crude oil, 25.6 mg.g^−1^ wet sediment), the common burrowing organism *Hediste (Nereis) diversicolor* and both the oil and *H. diversicolor*. The addition of *H. diversicolor* and its associated bioturbation did not affect the removal of petroleum hydrocarbons. After 270 days, 60% of hydrocarbons had been removed in all microcosms irrespective of the *H. diversicolor* addition. However, 16S-rRNA gene and 16S-cDNA T-RFLP and RT-PCR-amplicon libraries analysis showed an effect of the condition on the bacterial community structure, composition, and dynamics, supported by PerMANOVA analysis. The 16S-cDNA libraries from microcosms where *H. diversicolor* was added (oiled and un-oiled) showed a marked dominance of sequences related to *Gammaproteobacteria*. However, in the oiled-library sequences associated to *Deltaproteobacteria* and *Bacteroidetes* were also highly represented. The 16S-cDNA libraries from oiled-microcosms (with and without *H. diversicolor* addition) revealed two distinct microbial communities characterized by different phylotypes associated to known hydrocarbonoclastic bacteria and dominated by *Gammaproteobacteria* and *Deltaproteobacteria*. In the oiled-microcosms, the addition of *H. diversicolor* reduced the phylotype-richness, sequences associated to *Actinobacteria*, *Firmicutes* and *Plantomycetes* were not detected. These observations highlight the influence of the bioturbation on the bacterial community structure without affecting the biodegradation capacities.

## Introduction

Crude oils are complex mixtures of hydrocarbons, asphaltenes, resins, and associated metals, mainly vanadium and nickel [1], which may have serious environmental toxic effects. The biodegradation of hydrocarbons by microorganisms is a crucial process by which petroleum and other hydrocarbon pollutants are eliminated from the environment [2].

The fate of hydrocarbons in the environment is not only dependent on microbial activities, but also on sediment characteristics, physical-chemical factors (electron acceptors, temperature), nutrients, co-metabolite availability, and bioturbation [3]. In particular, bioturbation is created and aided by the benthic macrofauna which physically mixes sediments and introduces oxygen into sediments by burrow ventilation may greatly affects the biogeochemical cycles of nutrients [4], the fate of contaminants and the microbial metabolisms [4]. Several studies have demonstrated that bioturbation processes play an important role in the burial and the degradation of aliphatic hydrocarbons [5], PAHs [6] and acyclic hydrocarbons [7]. In addition, polychaetes, a class of marine annelid worms, have the enzymatic potential to solubilize aromatic compounds [8], [9]. Burrowing activity can also affect the overall microbial metabolic activities such as nitrifying [10] and sulfate-reducing activities [11], [12]. It has been shown that the increase in microbial abundances and activities is due to complex biogeochemical interactions induced by bioturbation [13]–[15]. Previous reports suggest that bioturbation by macrofauna drives microbial community structure and activities, which can also be affected by the presence of hydrocarbons. To the best of our knowledge, only one study reported specific bacterial patterns due to bioturbation activities in the presence of hydrocarbons [16]. The lack of studies is most likely due to the difficulty in creating experiments for studying natural bioturbated microbial communities subjected to oil pollution. To overcome these difficulties, different strategies have been developed by performing either *in situ* studies [7] or microcosm [16] studies using sediments from which macrofauna have been eliminated and then reintroduced. The former is not a suitable strategy to investigate the effect of a pollutant since *in situ* oil spiking requires special authorization and special technical means for oil containment, while the latter would modify the sediment structure. Our strategy was to increase bioturbation activity by adding polychaetes to bioturbated mudflat sediment in order to investigate how oil affects the bacterial community structure and composition. We hypothesized that the addition of polychaetes stimulates the bioturbation activity which could result in the selection of a particular bacterial community with an increased biodegradation capability. Thus, an original microcosm system maintaining the structure of muddy sediments under tidal cycles was set up in order to ensure conditions close to those in the natural environment. A set of chemical and microbial analyses was performed over a nine-month period to monitor i) the removal of petroleum by estimating hydrocarbon contents ii) the macrofaunal reworking activity and iii) the structures and composition of bacterial communities. This multidisciplinary approach, combined with multivariate analyses, showed that a specific bacterial community was obtained in response to oil addition in bioturbated sediments. The results presented here showed that polychaetes strongly affected bacterial community structures without changing oil degradation capacities.

## Materials and Methods

### Sampling Area

Mud sediments for microcosm experiments were collected in autumn 2007 at the tidal basin Aber-Benoît, located in the Brittany region (Treglonou, France, 48° 33′12.40′′N; 4° 32′8.69′′W).

This sediment showed a pH of 7.8 with an organic carbon content of 6.8% and a salinity of 35‰. The sediment moisture content was 60% and chemical analysis detected about 4 mg of biogenic hydrocarbons per gram of dry sediment in the upper layer of sediment (2 cm). Sediments were composed of 3% clay (<3.9 microns), 31% silt (3.9–62.5 microns) and 66% sand (62.5–2000 microns). The density and characterization of autochthonous macrofauna were determined from three cores collected from the sampling site. The macrobenthic community was composed of four species, the marine annelid worms *Nephtys hombergi* and *Hediste (Nereis) diversicolor*, the mollusk *Hydrobia ulvae* and the bivalve *Mya arenaria*, for an average total density of 2419±6.08 ind.m^−2^. More specifically, the gallery-forming polychaete *Hediste diversicolor* represented 61 g.m^−2^ (509 ind.m^−2^).

### Microcosm Setup

**Figure 1:**
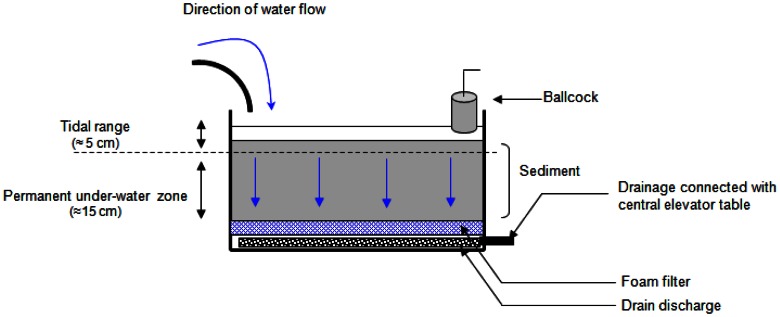
Microcosms’ box design. Sediments were maintained in such microcosm boxes and subjected to 12-hour tidal cycles, renewing the seawater with each tidal cycle.

Sediments were sampled with a core collector (20–25 cm sediment depth), and transferred while maintaining their integrity, over geotextile membrane into microcosm boxes (65 cm×50 cm×41 cm; approximately 30 L of sediment). Twelve microcosm boxes were set up and connected to a device supplying natural, sand-filtered, and UV-treated seawater from Oceanopolis aquarium (Brest, France), thus ensuring constant sea water quality. Tidal cycles (12 hours) were applied using a water level control consisting of an up and down drainage system. Seawater was renewed with each tidal cycle (20 L of water per microcosm box). At high tide, the water-level was maintained about 5 cm above the sediment surface using a faucet ballcock (Fig. 1). The microcosms were set up in a hall at room temperature (ranging from 10 to 20°C). They were not directly exposed to sunlight limiting evaporation and photodegradation of hydrocarbons. After 7 days of sediment stabilization, four conditions were applied in triplicate (N = 12) as follows: (i) CTRL: control condition, (ii) BAL: oil addition, (iii) NEREIS: addition of *H. diversicolor* and (iv) NEREIS+BAL, addition of oil and *H. diversicolor*. In order to standardize sediments, the 2 cm upper layer of each microcosm box was collected, pooled and mixed for homogenization. Half of the homogenized sediments were mixed with oil in order to obtain 25.6 mg of oil per gram of wet sediment. This oiled sediment was then spread in a 2 cm layer over the contaminated conditions (BAL and NEREIS+BAL) sediment surface. Thus, each oiled microcosms began the experiments with the same contamination level. We used Blend Arabian Light crude oil (BAL) which corresponds to a standardized Arabian crude oil, often used as model crude oil. The BAL oil used was distilled at 110°C to eliminate the more volatile hydrocarbon compounds; such oil is named BAL 110 oil (Arabian Light crude oil topped at 110°C). For the non-contaminated conditions (CTRL and NEREIS) the half un-oiled homogenized sediments was spread over the sediment surface (2 cm layer).

*Hediste (Nereis) diversicolor*, common polychaete worm found in mudflat sediments able to live within the first 10 cm of sediments, was added to the microcosms in order to increase burrowing activity and, thus, sediment reworking (NEREIS and NEREIS +BAL). Thus the *H. diversicolor* density was doubled (final density reaching 982 ind.m^−2^) but the biomass increased 10-fold (addition of 614 g.m^−2^), due to the addition of larger polychaete individuals.

Two hours after the experimental conditions were established, the tidal cycles were initiated. Subsamples of sediment were collected after 2, 7, 30, 90, 180 and 270 days of microcosm incubation (November 2007–July 2008). For chemical analyses, one core sample (Falcon tube, 2.5 cm×8 cm) was collected for each microcosm (triplicates per condition). For molecular analyses, superficial samples (0–2 cm, Eppendorf tube, 2 mL) were collected in duplicate for each microcosm (sampling replicate). Thus, six replicates per condition at each sampling time were obtained. The sub-samples were collected 10 min. after reaching “low tide” randomly in order to consider the sediment’s heterogeneity; the objective being to analyze the bulk sediments. The sampling hole was plugged with a plastic tube in order to maintain the sediment’s structure. Samples were quickly frozen in liquid Nitrogen and stored at −80°C until analyzed at the end of the experiment when all samples were collected. It is important to note that the pH was stable around 7.5 during the experiments; there was no significant variation between high tides and low tides.

### Hydrocarbon Extraction and Analysis

Prior to analyses, each sediment core was divided into four layers (0–2/2–4/4–6/6–8 cm). Each layer was then split for the determination of moisture content (50°C for 24 hours), total petroleum hydrocarbon (TPH) concentrations and alkanes/aromatics analyses.

Sediment samples were spiked with deuterated aliphatic (d_42_-eicosane) and aromatic hydrocarbons (d_8_-naphthalene, d_10_-biphenyl, d_10_-phenanthrene, d_12_-chrysene and d_12_-benzo(a)pyrene) surrogate standard (LGC standard, France) and then extracted with 30 mL methylene chloride at 100°C and 2000 psi for 14 min in a Dionex ASE 200 accelerated solvent extractor. Extractions of sediment were performed twice for each sample. The organic extracts were dried over Na_2_SO_4_ (activated at 400°C for 4 hours) and concentrated to approximately 2 mL using a Syncore (Büchi, Germany). Extracts were fractionated using a SPE (Solid Phase Extraction) cartridge (silica/cyanopropyl (SiO2/C3-CN; 1.0/0.5 g, 6 mL; Interchim, France) [17]. Saturate and aromatic fractions were eluted simultaneously with 8 mL of methylene chloride/pentane (80:20, v/v) and concentrated to approximately 2 mL.

TPH contents were analyzed according to the standard NF EN ISO 9377-2 protocol using high temperature gas chromatography with a flame ionization detector (HTGC/FID) HP 6890N equipped with an autosampler. 1 µL of sample was injected in purged splitless mode (splitless time: 2 minutes, flow: 50 mL/min) at 425°C. The oven temperature was initially 30°C, then was increased at 20°C per minute 1 minute after injection to a final temperature of 415°C, then held for 30 minutes. The metallic chromatographic column was a DBHT-SIMD (Agilent J&W): 5 m×0.53 mm ID×0.15 µm film thickness. The helium carrier gas was maintained at a constant flow of 15 mL/min. Different concentrations of BAL 110 diluted with methylene chloride were used to calibrate the method and then to quantify the TPH extracted from the sediment samples.

Alkanes and aromatics were quantified using gas chromatography coupled with mass spectrometry (GC/MS). The GC/MS was an HP 6890N equipped with a split/splitless injector (Splitless time: 1 min, flow 50 mL/min) coupled to an HP 5973 Mass Selective Detector (MSD) (Electronic Impact: 70 eV, voltage: 1200 V). The injector temperature was maintained at 300°C. The interface temperature was 300°C. The GC/MS temperature gradient was from 50°C (1 min) to 300°C (20 min) at 5°C/min. The carrier gas was Helium at a constant flow of 1 ml/min. The capillary column used was an HP-5 MS: 30 m×0.25 mm ID×0.25 µm film thickness. n-Alkane and PAH semi-quantifications were performed using Single Ion Monitoring (SIM) mode with the most representative fragment (saturates) or the molecular ion (PAHs) of each compound at a minimum of 1.4 cycles/s.

The TPH concentrations were tested by a three-way analysis of variance (ANOVA) in order to investigate the effects of time (T), the addition or non-addition of *H. diversicolor* (N), the depth (D) and all their interaction with the TPH contents of the microcosm boxes. A prior analysis was run to test the dependence of temporal observations (*i.e* repeated measures) with a linear mixed model with T,N,D and their interactions as fixed factors and the temporal pseudo-replication specified as random effect (using the package nlme in R, [18]). However, no temporal autocorrelation was detected and only the fixed factors were retained in the three-way ANOVA. Prior to statistical analysis, homoscedasticity was assessed by Hartley's Fmax test and data was log transformed in order to meet the test assumptions. Significant differences between means of groups were determined using a Duncan’s pairwise multiple comparison test. The significance level was set at α = 0.05. Statistical data analyses were performed with STATISTICA software, version 5.5 (StatSoft, USA) and the R software (version 2.11.1).

### Reworking Activity

The sediment reworking activity in the different experimental microcosm boxes was estimated using fluorescent particulate tracers (luminophores; [19]). One hour after the introduction of *H. diversicolor* into the NEREIS and NEREIS-BAL microcosm boxes, a suspension of 100 g of orange luminophores (63–125 µm) was homogeneously spread over the sediment surface of each experimental microcosm box. At the end of the experiment, one sediment core (I.D.: 10 cm, length: 15 cm) was sampled from each experimental microcosm box. Each core was then sectioned in 0.5-cm thick layers from the surface down to 2 cm depth, then in 1-cm thick layers down to 10 cm. The sediment from each layer was removed separately and sieved through a 250 micrometer mesh to remove macrofauna from sediment particles. Sediments were then freeze-dried, gently crushed to powder and homogenized. Sediment subsamples were taken for luminophore counting under UV-light (digital camera Olympus C-2500L; image analysis software Image-Pro Plus). This resulted in a particulate tracer distribution profile over depth, for each sediment core. Then, sediment reworking (i.e. production of a biodiffusion-like coefficient *Db* describing particle transport in the whole sedimentary column) was quantified by applying a one-dimensional diffusion model (script developed in Matlab®) to the data [20].

### Total DNA Isolation of Microbial Communities

DNA was extracted from the 136 samples collected in the upper layer of sediment (2 cm, 250 mg) using the UltraClean Soil DNA kit (MoBio Laboratories) according to the manufacturer’s recommendations. The protocol was modified as follows: sediment was centrifuged at 5000× *g* for 15 min, to remove the water phase; the initial step of horizontal vortexing was performed for 30 min. DNA was eluted in 50 µL water and stored at −20°C.

### RNA Isolation and Reverse Transcription

Total RNA extractions were performed from the 136 samples collected in the upper layer of sediment (2 cm, 2 g) using the RNA Power Soil kit (MoBio Laboratories) according to the manufacturer’s recommendations. Only RNase-free certified plastic-ware was used and all solutions were prepared with sterile diethyl pyrocarbonate-treated (DEPC) water. DNA was removed from the total nucleic acid extraction by DNase digestion, using Turbo DNA-free™ protocols (Ambion). The complete removal of DNA was verified by a control PCR using primers of bacterial 16S rRNA directly on RNA extract (PCR conditions described below) and the RNA quality was controlled by capillary electrophoresis (Agilent Bioanalyzer). A concentration of 0.5 µg of DNase-treated RNA (Turbo DNase; Ambion) was reverse-transcribed with Moloney murine leukemia virus reverse transcriptase (M-MLV RT; USB Corporation) as previously described [21] with minor modifications including the use of random hexamers (5 µL at 0.1 U A260; Roche) and an incubation time of 2 h at 42°C. The RT products were used immediately for PCR amplifications and the remaining products were stored at −20°C. Possible DNA contamination of RNA templates was monitored by PCR amplification of RNA aliquots without a reverse transcription step. No DNA was detected in these reactions.

### Amplification of Bacterial 16S rRNA Gene

16S rRNA genes were amplified from the 136 nucleic acid samples using the primers 63f (5′-CAGGCCTAACACATGCAAGTC-3′) and 1387r (5′-GGGCGGWGTGTAACAAGGC-3′) specific for Bacteria [22]. The forward primer was labeled at the 5′ end with phosphoramidite fluorochrome carboxyfluorescein (FAM). PCR conditions were as follows: initial denaturation (94°C for 3 min) followed by 35 cycles of denaturation (94°C for 30 s), annealing (58°C for 30 s), and extension (72°C for 60 s) and a terminal extension (72°C for 10 min). The reaction mix (50 µL final volume) contained 50 mM buffer, 0.4 mM dNTP, 0.2 µM of each primer, 1.25 U of Taq polymerase (Ozyme) and 10 ng of DNA template. PCR products were visualized by agarose gel electrophoresis and purified with the PCR purification kit (GE Healthcare).

### T-RFLP Analysis

Purified 16S rRNA amplicons were separately digested by 3 U of HaeIII and HinfI restriction enzymes at 37°C (New Englands Biolabs) in a final volume of 10 µL for 3 h. A desalting step by dialysis was performed using a 0.022 µm pore filter (Millipore). Then, digested products (1 µL) were mixed with deionised formamide (10 µL) and 0.25 µL of internal size standard (TAMRA GS-500, Applied Biosystems). The samples were denatured by heating at 95°C for 5 min and then immediately transferred onto ice. Fluorescently labeled fragments were separated and detected with an ABI PRISM 310 capillary sequencer (Applied Biosystems) run in GenScan mode. Injection was performed electrokinetically at 15 kV for 30 s and the runs at 15 kV were completed within 30 min. T-RFLP profiles were analyzed using GENSCAN version 3.1 software (Applied Biosystems). T-RFLP profiles were normalized by calculating relative abundances of each peack from height fluorescence intensity. Only terminal fragments whose size ranged from 35 bp to 500 bp and whose height was greater than 30 fluorescence units were considered for analysis.

The comparison of DNA− and RNA-based T-RFLP profiles was used to determine the active changes in the bacterial communities. For each sample, we calculated the percentage of OTUs that were present in both fractions (DNA+ RNA+), those were present only in DNA-based fractions (DNA+ RNA−) and those only present in RNA-based fractions (DNA− RNA+). Relationships between the different fractions (RNA+DNA+, RNA−DNA+, RNA+DNA−) and hydrocarbon concentrations (TPH, n-alkanes and PAHs) across time were explored with simple linear regressions (lm function in R software).

### Statistical Comparison of T-RFLP Profiles

The T-RFLP profiles were standardized by Dunbar’s method [23]. Then, raw data were normalized by dividing each peak height by the total signal intensity of the corresponding profile. To deal with pseudo-replication within microcosm box (two sampling replicates within each microcosm box), the average over the pseudo-replicates were calculated in order to obtain three profile replicates per condition (biological replicates, BRs). The dissimilarities among BRs were estimated with a Bray-Curtis distance (Table S1 in the supplemental material). The effects of time, BAL addition and NEREIS addition on relative abundance of DNA Operational Taxonomic Units (OTUs) and cDNA OTUs in communities were tested with permutational multivariate analysis of variance (PerMANOVA; [24]). We performed a four-way PerMANOVA with time, BAL effect (BAL addition or not), and NEREIS effect (NEREIS addition or not) and BRs as additional factor included to account for repeated units. To assess this model, permutations were constrained within BRs, so that the effects of time and its interactions with other factors were taken into account (model I in Table S3 in the supplemental material). We also ran a three-way factorial PerMANOVA with BAL effect, NEREIS effect and their interaction and with time as an additional factor. In this model, permutations occurred within each sampling time in order to obtain a proper estimation of the BAL and NEREIS effects (model II in Table S3 in the supplemental material). These analysis were carried out with the *adonis* function in the *vegan* R package, [25] and based on untransformed abundance data, using a Bray Curtis distance and 999 random permutations (α = 0.05). Since most of terms (main factors and their interactions) of PerMANOVA model I and II were significant, two dimension non-metric multidimensional scaling (nMDS) ordinations (based on Bray Curtis distance) were used to visually represent dissimilarities between conditions at each sampling time and between sampling time for each conditions. The robustness of nM-MDS analysis was evaluated by calculating Kruskal Stress. Instead of performing Duncan post hoc pairwise comparisons, we determined the most similar communities across time and conditions with a hierarchical analysis performed with the average profiles from the three BR profiles. The groups were formed according to the similarity obtained between the BRs and the significant differences between groups were tested with an analysis of similarities (ANOSIM). Hierarchical clustering, nMDS analysis and ANOSIM were performed with Primer 6 software (Primer E, Plymouth, UK).

### 16S cDNA Library Construction and Analysis

16S cDNA libraries were obtained with unlabeled PCR products from 270 days for the four conditions (CONTROL, NEREIS, BAL, NEREIS+BAL). PCR products were obtained from the six replicates per condition and pooled before cloning. The pooled PCR products were cloned in *Escherichia coli* TOP10F’ using the pCR2.1 Topo TA cloning kit (Invitrogen Inc.). The presence of insert DNA was checked by PCR using M13 primers (Eurogentec) surrounding the cloning site. For each cDNA library, 100 clones were taken randomly for sequence determination. The clones were sequenced by GATC Biotech SARL (Konstanz, Germany). The sequences were compared with the GenBank nucleotide database library by BLAST on-line searches [26]. The presence of chimeric sequences was checked with Mallard and Pintail tools. Multiple sequence alignment was performed using the CLUSTALX [27]. Phylogenies were constructed with the MOLECULAR EVOLUTIONARY GENETICS ANALYSIS v3.0 program [28] using the Kimura two-parameter model and the neighbor-joining algorithm. The significance of branching order was determined using bootstrap analysis with 1000 resampled data sets. The DOTUR program was used to determine Operational Taxonomic Units (OTUs) defined as sequence groups in which sequences differed by 3% for *16S rRNA* sequences [29]. PAST (Paleontological Statistics v1.60) software was used to perform rarefaction analysis and calculate diversity indexes for each 16S cDNA library. The coverage value is given as C = 1−(n1/N) where n1 is the number of clones which occurred only once in the library [30] and species richness was calculated using the Chao Estimator website (http://www2.biology.ualberta.ca/jbrzusto/rarefact.php) [31]. The compositions of bacterial communities were compared by clustering analysis using Pearson’s correlation as a similarity distance and a heatmap was drawn with gplots package in R software [32]. *In silico* restrictions of clone sequences were performed using the Mobyle website (http://mobyle.pasteur.fr/cgi-bin/portal.py) [33].

### Nucleotide Sequence Accession Numbers

The sequences determined in this study have been submitted to the Genbank database under accession Nos. JF774416 to JF774826.

## Results

### Characterization of the Biological Reworking Activity

The abundance of *H. diversicolor* was maintained throughout the experiment, and was not significantly affected by oil addition (NEREIS: 1782±382 ind.m^−2^; NEREIS+BAL: 2334±989 ind.m^−2^; p-value = 0.26). Biodiffusion-like coefficients (*Db*) varied according to the experimental conditions. The microcosms to which *H. diversicolor* were added showed higher *Db* (NEREIS: 0.70±0.08 cm^2^.y^−1^; Mean ±SD; *n* = 3) compared to the other microcosms (*Db* from 0.18 to 0.33 cm^2^.y^−1^) indicating a marked increase in burrowing with the addition of both *H. diversicolor* and oil (NEREIS+BAL: *Db* = 1.07±0.12 cm^2^.y^−1^; Mean ±SD; *n* = 3). This observation indicated that the reworking activity was enhanced by the oil addition. The luminophore distribution indicated that most of the reworking activity took place within the top 2 cm (see Fig. S1 in the supplemental material). We thus analyzed microbial community in the top 0–2 cm layer, given this luminophore distribution, and because it is the most oxygenated zone where efficient hydrocarbon degradation is expected.

### Fate of Petroleum Compounds

**Figure 2:**
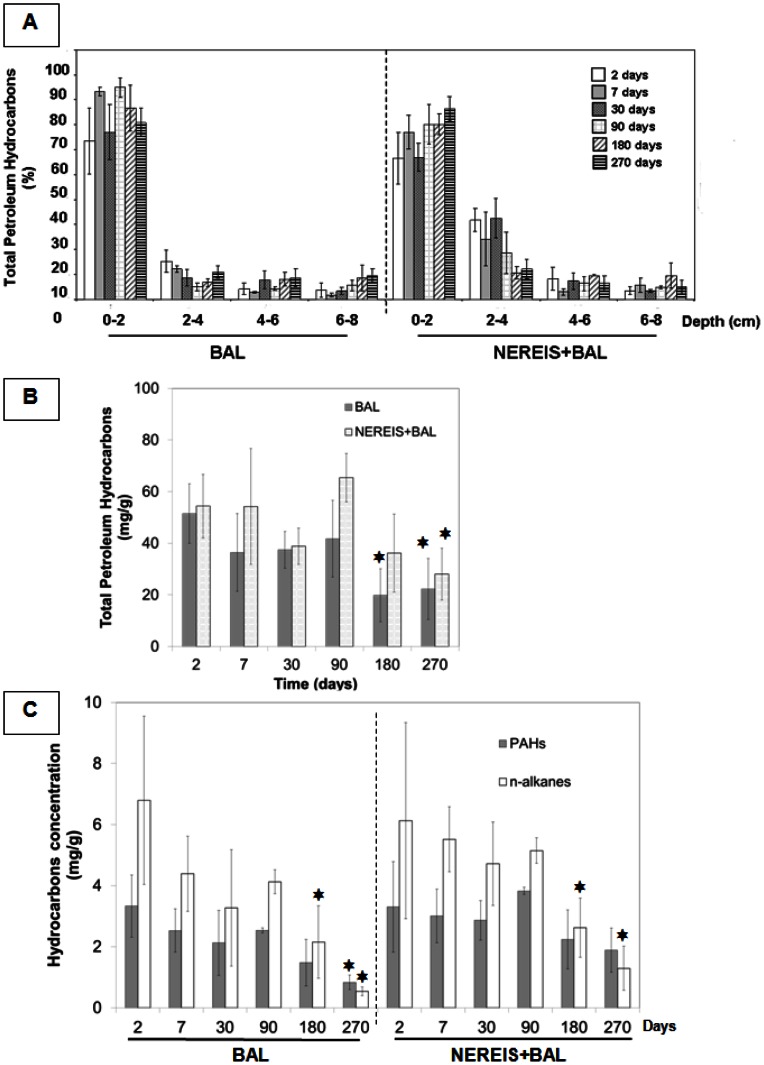
Chemical analyses of hydrocarbon content in sediments. A) Depth distribution of relative TPH content (%) in the BAL and NEREIS+BAL conditions during the 270 days of the experiment. The 100% TPH represents the total TPH per sampling time (addition of all depths). B) Total petroleum hydrocarbons (TPH) content during the 270 days of the experiment for the BAL and NEREIS+BAL conditions in the 0–2 cm layer. C) Concentration (µg/g of dry sediment) of n-alkanes and PAHs (from benzothipohene and its alkylated forms to chrysenes and their alkylated forms) during the 270 days of the experiment for the BAL (left) and NERREIS+BAL (right) conditions. The stars show significant differences obtained by the Duncan test (p-value<0.05) compared to the day 2 value.

In order to assess the fate of BAL 110 oil, the total petroleum hydrocarbon (TPH) content was monitored during the 270 days of the experiment (Fig. 2A, 2B). The distribution of TPH content according to depth highlighted the burrowing activity of *H. diversicolor,* particularly at an early stage when statistically significant differences between BAL and NEREIS+BAL conditions were observed for the 2–4 cm layer (p-value<0.05) (Fig. 2A). Indeed, in the polychaete bioaugmented condition (NEREIS+BAL), a mean of 23% of TPH was extracted from the 2–4 cm layer while the TPH content represented only 10% in the microcosm without *H. diversicolor* addition (BAL). The increase in the macrobenthic population and its related reworking activity clearly allowed deeper burying of TPH into the sediments.

The TPH concentration significantly varied with the time, the NEREIS addition, the depth and the different interactions among these factors (Table S2 in the supplemental material). According to the Duncan post-hoc test, the TPH concentration was significantly reduced irrespective of conditions after 90 days of incubation (BAL p-value = 0.0062; NEREIS+BAL p-value = 0.0048). By the end of incubation, 60% of the initial TPH had been removed (0–8 cm depth, see Fig. S2 in the supplemental material). Considering the 0–2 cm layer (Fig. 2B), no statistically significant difference was observed between the BAL and NEREIS+BAL conditions. 57% of the initial TPH was removed in the BAL condition and 48% was removed in the NEREIS+BAL condition. This result indicated that, throughout the 0–8 cm layer, an average of 87% of TPH removal was located within the 0–2 cm layer in both oil-contaminated conditions.

Focusing on the chemical composition of the biodegradable fraction (*n*-alkanes and PAHs; Fig. 2C), the *n-*alkane and the PAH contents significantly varied with time and the NEREIS addition (Table S2 in the supplemental material). The *n*-alkane content significantly reduced from day 180 (according to the Duncan test, p-value<0.05) in the BAL (68% at 180 days, 92% at 270 days) and NEREIS+BAL (57% at 180 days, 80% at 270 days) conditions, while PAHs were depleted at the end of the experiment only in the BAL condition (75% removal). In our experimental system, the removal of hydrocarbons could be explained by physical factors such as washing out due to the constant input of seawater for tidal simulation. However, we assume that washing out was a limited phenomenon because hydrocarbons were present until the end of the experiment (40% TPH at T_270d_), and that PAHs (16 PAH of the EPA list) were detected only at T_90d_ at 0.1 µg/L on the weekly survey of the discarded water. Furthermore, it is important to note that the *n*-C_18_/phytane ratio (Fig. S3 in the supplemental material) decreased progressively from T_90d_ (2.2±0.1 for BAL; 2.1±0.2 for NEREIS+BAL) to T_270d_ (1.0±0.1 for BAL; 0.7±0.4 for NEREIS+BAL) with significant differences according to the Duncan test (p-value<0.05). This observation indicated that a biodegradation process was involved in alkane removal since linear alkanes (such as *n*-C_18_) are more sensitive to microbial degradation than alkylated alkanes (such as phytane) [34].

### Dynamics of Bacterial Communities

**Figure 3:**
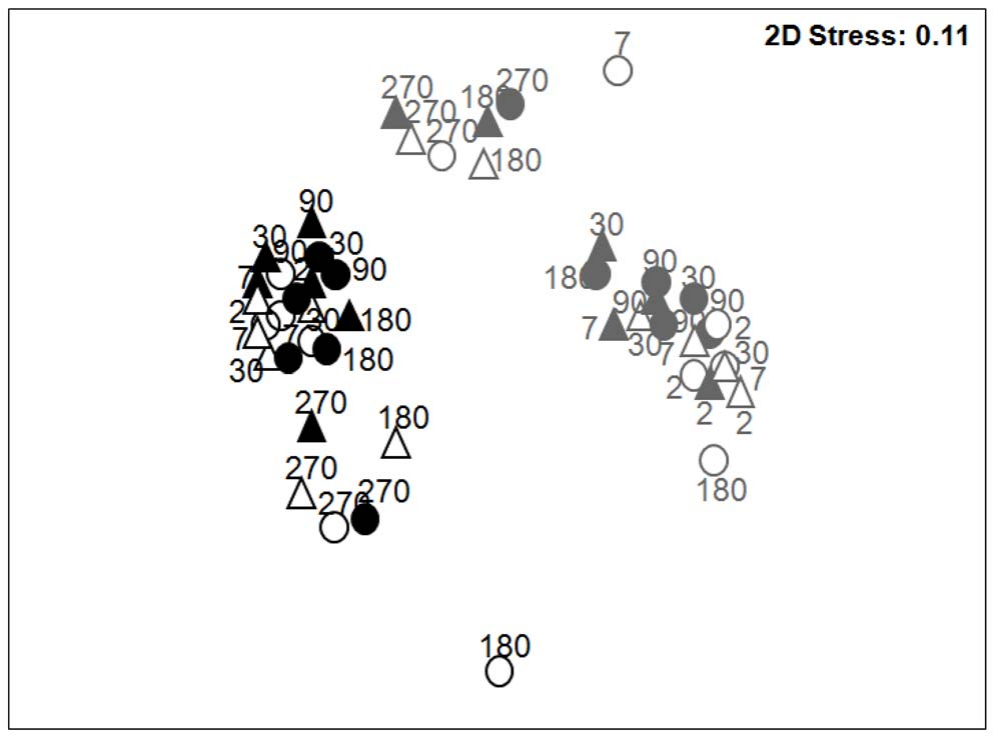
Bacterial community structure. nMDS analysis based on T-RFLP profiles (HaeIII and HinfI enzymes) from 16S rRNA gene (black) and 16S rRNA transcripts (gray) from microcosm sediment sampled at day 2, 7, 30, 90, 180 and 270 of incubation. CTRL: blank circle; BAL: filled circle; NEREIS: blank triangle; NEREIS+BAL: filled triangle.

**Figure 4:**
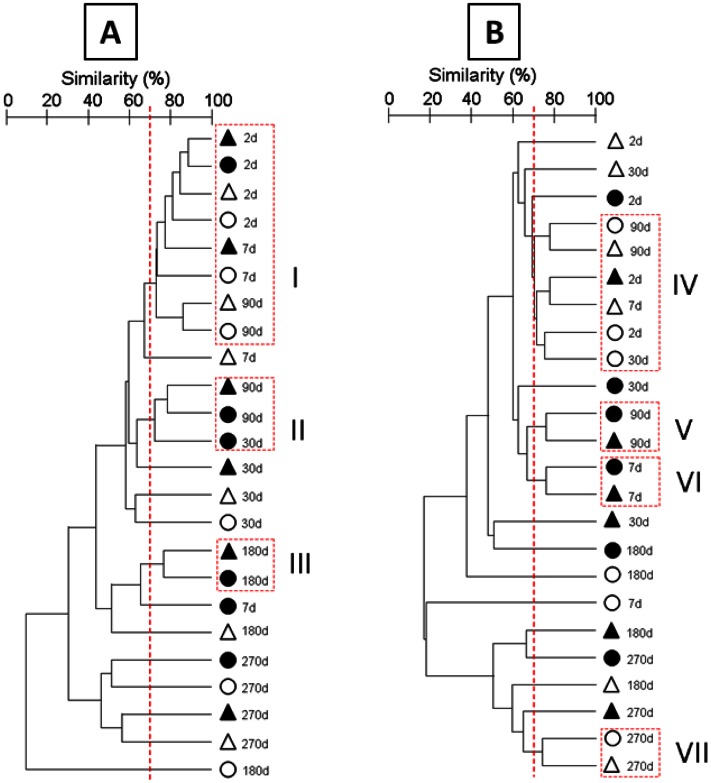
Bacterial community structure changes based on the 16S rRNA gene (A) and transcripts (B) for 270 days for the four conditions. Hierarchical analysis was performed using all replicates but only the average similarity between replicates is represented. CTRL, white circle; BAL, black circle; NEREIS, white triangle; NEREIS+BAL, black triangle. Clusters were formed under the 69.9% threshold.

Bacterial community structures were monitored by T-RFLP based on 16S rRNA gene (DNA) and transcript (cDNA) analyses for all incubation conditions throughout the experiment (Fig. 3). Pearson’s correlation analysis showed about 70% similarity between the replicates, revealing the spatial heterogeneity within and between microcosms, highlighting the importance of the sampling strategy to obtain representative samples (variability of the BRs is shown by nMDS analysis; Fig. S4A and B in the supplemental material). DNA and cDNA community structures were clearly separated by nMDS analysis (Fig. 3) but similar trends were observed, particularly across time. The bacterial DNA and cDNA community structures significantly shifted as function of time (model I PerMANOVA, Table S3 in the supplemental material), with a pronounced shift of both DNA and cDNA bacterial community structures from day 180 (Fig. 3). Despite the strong effect of time, the BAL addition and the NEREIS addition also significantly influenced the DNA and cDNA community structures (see model II PerMANOVA, Table S3 for a proper estimation of BAL and NEREIS effects). However, the significant three-level interaction (model I, Table S3 in the supplemental material) revealed complex shifts across time and conditions. Due to these complex interactions, the most similar communities across time and conditions were characterized by hierarchical clustering analysis (HCA). According to the similarity observed between biological replicates (BR), thresholds of 69.9% and 70.3% were applied to determine the cluster for 16S rRNA gene and transcript analyses respectively. The analysis of the total bacterial community (DNA analyses) revealed three significantly different clusters (Fig. 4A; see ANOSIM test values in Table S4 in the supplemental material), with cluster I containing samples from the beginning of the experiment (T_2d_) irrespective of the experiment conditions, and clusters II and III containing oiled samples from T_90d_ and T_180d_ respectively. This observation showed that, irrespective of *H. diversicolor* addition, the presence of oil determined a different bacterial community structure (BCS). Thus, in oiled microcosms, BCS at T_90d_ and T_180d_ were not affected by the addition of *H. diversicolor*, despite the pronounced effect observed in NEREIS treatment (180 days). Nevertheless, in NEREIS and BAL microcosms, BCS modifications were observed from day 7 of incubation, whereas in NEREIS+BAL condition, BCS modification was observed from day 30. At the end of the experiment, specific BCS were observed for each treatment showing a *H. diversicolor* effect in the presence of oil (T_270d_). Bacterial community dynamics are also presented in nMDS by incubation condition and over time (Fig. S4 and S5 in the supplemental material). In the BAL and NEREIS+BAL conditions, the most pronounced modification of the community structure was observed at the end of the experiment (T_270d_), testifying to the impact of oil on BCS. nMDS also shows the variability between microcosm replicates, represented by confidence ellipses that take into account the standard deviation of replicates. Although the predictive identification of OTUs has to be taken with caution, the *in silico* restriction of cloned sequences allowed clear identification at the phylum/class taxonomic level (see Fig. S6 in the supplemental material). At T_180d_, BAL and NEREIS+BAL communities were mainly composed of OTUs belonging to *Gammaproteobacteria* representing about 45% of the profiles, explaining their similarity. The shift between T_180d_ and T_270d_ was characterized by a modification of the composition of OTUs related to *Gammaproteobacteria*, especially in the NEREIS+BAL community.

With the cDNA analyses (Fig. 4B and Table S4 in the supplemental material), the HCA confirmed the BCS shifts observed by the DNA analyses further highlighting the treatment effects. Nevertheless, in contrast to the BCS shifts observed through DNA analyses, oiled bacterial communities (BAL and NEREIS+BAL) clustered together from day 7 (Fig. 4B, clusters V and VI) revealing a fast response of the bacterial community to oil addition. After 90 days, distinct BCS were observed except at T_270d_, with un-oiled samples (CTRL and NEREIS) forming a cluster (cluster VII). Finally, at the end of the experiment (T_270d_), BCS were clearly distinct from T_2d_, as also observed in nMDS represented by incubation conditions (Fig. S4B in the supplemental material). Furthermore, *in silico* digestion of cloned sequences by restriction enzymes (see Fig. S6 in the supplemental material) revealed different taxonomic patterns to those observed with the DNA analyses, particularly the OTUs related to *Deltaproteobacteria* that were found in high abundance, whereas they were not detected by the DNA analyses. At T_180d,_ their abundance in the BAL community (21.4%) compared to the NEREIS+BAL community (2.4%) explained the discrepancies observed between these communities. At T_270d_, the NEREIS+BAL community exhibited a higher abundance of OTUs related to *Gammaproteobacteria* (56.3%) while the *Alphaproteobacteria* were more abundant in the BAL community (19.5%) than in the NEREIS+BAL community (2.3%).

**Figure 5:**
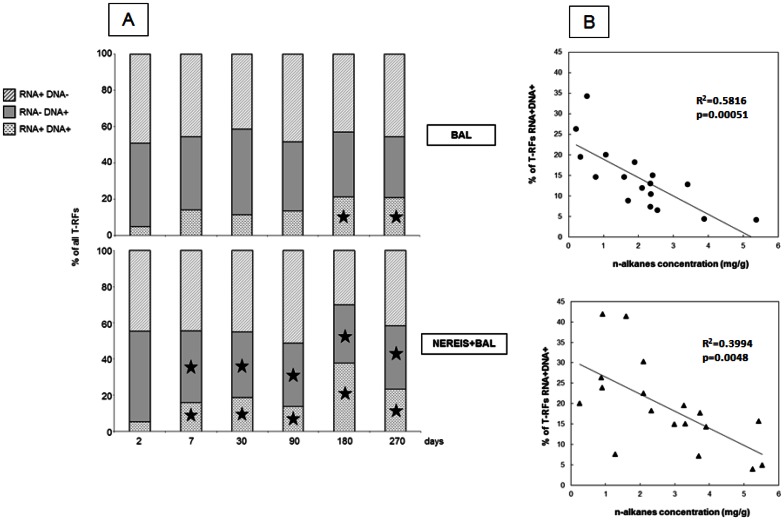
Comparison of 16S rRNA genes and 16S rRNA transcripts based on T-RFLP analyses from BAL and NEREIS+BAL. (**A**) Percentage of Operational Taxonomic Units (OTUs) specific to the 16S rRNA genes (RNA− DNA+) or the 16S rRNA transcripts (RNA+ DNA−), or common to both (RNA+ DNA+). The stars show significant differences obtained by the Duncan test (p-value<0.05) compared to the day 2 value. (B) Relationships between percentage of OTUs common to 16S rRNA genes and transcripts (RNA+ DNA+) with n-alkanes concentrations.

The comparison of T-RFLP analyses of 16S rRNA genes and 16S rRNA transcripts (Fig. 5A) showed that the active population captured at the genomic level (RNA+ DNA+) in BAL and NEREIS+BAL conditions represented approximately 20% at T_270d_. However, during the course of the experiment, different trends were observed according to the condition. The OTUs common to DNA and RNA analyses (RNA+ DNA+) increased significantly from day 180 in the BAL condition. In the NEREIS+BAL condition, it increased from day 7, with a concomitant decrease of specific OTUs observed through the DNA analyses (RNA− DNA+). These observations suggested an early effect when *H. diversicolor* was added.

The OTUs common to both fractions (RNA+ DNA+) were significantly and negatively correlated with the n-alkanes concentrations (Fig. 5B). The same results were obtained with TPH and PAHs concentrations (data not shown). The fractions of OTUs present only in DNA-based analysis (RNA− DNA+) and only in RNA-based analysis (RNA+ DNA−) were not significantly related with the hydrocarbon concentrations. These observations indicated that the bacterial active dominant fraction increased concomitantly with hydrocarbon biodegradation.

### Bacterial Community Composition by 16S rRNA Transcript Analyses

In order to characterize the bacterial community compositions at T_270d_, after hydrocarbon removal was observed, 16S cDNA libraries were constructed for each condition (see Fig. S7 to S9 in the supplemental material). The characteristics of the libraries are shown in Table 1. The coverage of the T_270d_ libraries from 51.4 to 76.4% indicated that the libraries were representative of the most abundant populations. The analysis of 333 complete sequences, representing 186 OTUs (considering a 97% similarity threshold), showed that 80.9% of OTUs were found specifically in a single condition, which supported the LIBSHUFF library’s comparison, revealing that the four libraries were composed of significantly different OTUs (p-values between 0.001 and 0.004).

**Table 1:** Characteristics of the CTRL_270d_, BAL_270d_, NEREIS_270d_ and NEREIS+BAL_270d_ cDNA libraries.

	CTRL_270d_	BAL_270d_	NEREIS_270d_	NEREIS+BAL_270d_
Number of clones	88	82	73	90
Number of OTUs	41	49	52	44
Chao estimation	85±19	90±16	93±15	64±9
% of coverage	73.9	63.4	51.4	76.4
Simpson index	0.953	0.971	0.977	0.965
Shannon index	3.41	3.726	3.865	3.574

**Figure 6:**
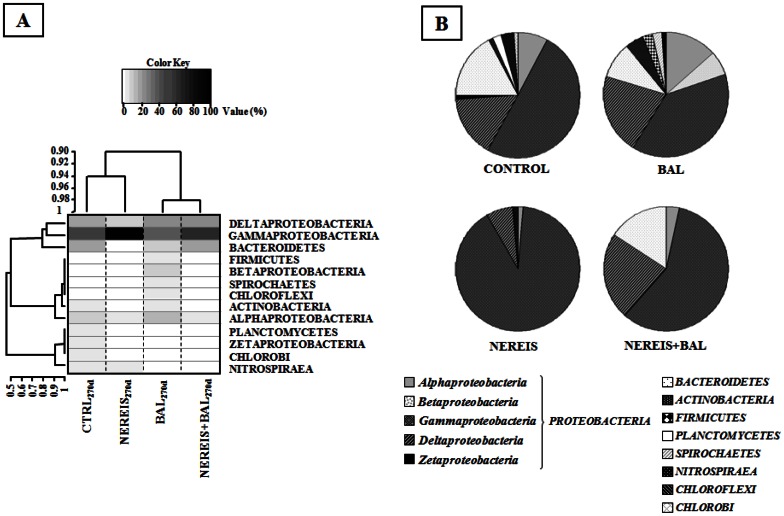
Relationship between microcosm conditions and bacterial phylotypes (phylum level). (A) Heatmap with double clustering. The microcosm conditions are clustered at the top and bacterial phylotypes are clustered on the left of the heatmap according to their intensity profile similarity. Black corresponds to a higher abundance and white to an absence of taxa. (B) Relative abundance (%) of phylogenetic groups in 16S rRNA libraries from the four incubation conditions at 270 days.

The comparison of the libraries’ composition at the phylum level highlighted the differences between the bacterial communities (Fig. 6, Table 2). Sequences related to *Proteobacteria* and *Bacteroidetes* being the most abundant in all libraries except the NEREIS library (T_270d_), in which only *Proteobacteria* (98.6%) and *Nitrospirae* (1.4%) were detected. Among the *Proteobacteria,* the *Gammaproteobacteria* was the most abundant class found, especially in the NEREIS library (90.3% of sequences). *Deltaproteobacteria*, the second most abundant *Proteobacteria*, were found with similar abundances (20.7% and 22.5%) in the oiled NEREIS+BAL and BAL libraries, while they represented only 6.9% in the NEREIS library. Interestingly, in agreement with T-RFLP analyses, *Alphaproteobacteria* were found to be most abundant in the CTRL and BAL libraries (7.9% and 13.4% respectively) while they represented less than 4% in libraries from microcosms in which *H. diversicolor* was present. *Betaproteobacteria* were found only in the BAL library and one sequence related to *Zetaproteobacteria* was found in the CTRL library.

**Table 2:** Identification of phylotypes from sequence analyses of cDNA libraries and their relative abundance (%).

Phylogenetic groups			CTRL_270d_	BAL_270d_	NEREIS_270d_	NEREIS+BAL_270d_	
**ALPHAPROTEOBACTERIA**			**7.9**	**13.4**	**1.4**	**3.3**	
	Rhodobacteraceae		2.3 (1)	2.4 (1)	–	2.2 (2)	
	Rhodospirilaceae		2.3 (2)	–	–	1.1 (1)	
	Sphingomonadaceae		–	–	–	–	
	Erythrobacteraceae		1.1 (1)	–	–	–	
	Hyphomicrobiaceae		–	–	1.4 (1)	–	
	Bradyrhizobiaceae		–	2.4 (2)	–	–	
	Rhodobiaceae		1.1 (1)	–	–	–	
	Methylocystaceae		–	2.4 (1)	–	–	
	Unclassified		1.1 (1)	6.2 (2)	–	–	
**BETAPROTEOBACTERIA**			**–**	**6.2**	**–**	**–**	
	Rhodocycleae		–	–	–	–	
	Comamonadaceae		–	1.2 (1)	–	–	
	Burkholderiaceae		–	5 (1)	–	–	
**GAMMAPROTEOBACTERIA**			**50**	**39.3**	**90.3**	**58.4**	
	Alteromonadaceae		7.8 (4)	7.4 (4)	16.7 (10)	20.3 (9)	
	Chromatiaceae		9.2 (4)	1.2 (1)	9.7 (5)	3.4 (1)	
	Ectothiorhodospiraceae		–	3.7 (1)	1.4 (1)	–	
	Unclassified Chromatiales		1.1 (1)	2.4 (2)	1.4 (1)	5.6 (2)	
	Unclassified Oceanospirillales		–	–	1.4 (1)	–	
	Piscirickettsiaceae		–	–	–	1.1 (1)	
	Pseudomonadaceae		–	–	–	5.6 (1)	
	Colwelliaceae		–	–	–	–	
	OM Group		–	2.4 (1)	1.4 (1)	–	
	SOB		31.9 (9)	19.8 (8)	56.9 (26)	21.3 (9)	
	Unclassified		–	2.4 (1)	1.4 (1)	1.1 (1)	
**DELTAPROTEOBACTERIA**			**16**	**20.7**	**6.9**	**22.5**	
	Desulfobacteraceae		–	15.9 (8)	5.5 (3)	13.5 (7)	
	Desulfobulbaceae		1.1 (1)	2.4 (1)	–	–	
	Nitrospinaceae		1.1 (1)	–	–	–	
	Desulforomonadaceae		–	1.2 (1)	1.4 (1)	–	
	Sorangiineae		9.2 (1)	–	–	1.1 (1)	
	Halangiaceae		1.1 (1)	–	–	–	
	Unclassified		3.5 (1)	1.2 (1)	–	7.9 (1)	
**ZETAPROTEOBACTERIA**			**1.1**	**–**	**–**	**–**	
	Mariprofundaceae		1.1 (1)	–	–	–	
**BACTEROIDETES**			**17.1**	**9.6**	**–**	**15.8**	
	Flavobacteriaceae		–	2.4 (2)	–	1.1 (1)	
	Cytophagaceae		17.1 (6)	3.6 (1)	–	14.7 (7)	
	Sphingobacteraceae		–	–	–	–	
	Flexibacteraceae		–	2.4 (1)	–	–	
	Unclassified		–	1.2 (1)	–	–	
**ACTINOBACTERIA**			**1.1**	**4.8**	**–**	**–**	
	Propionibacteraceae		–	1.2 (1)	–	–	
	Geodermatophilaceae		–	–	–	–	
	Unclassified		1.1 (1)	–	–	–	
**FIRMICUTES**			–	**2.4**	–	–	
	Veillonellaceae		–	1.2 (1)	–	–	
	Clostridiaceae		–	–	–	–	
	Unclassified	–	–	–	–	–	
**PLANCTOMYCETES**			**2.2**	–	–	–	
	Planctomycetaceae		2.2 (1)	–	–	–	
**DEFERRIBACTERES**			–	–	–	–	
	Unclassified	–	–	–	–	–	
**SPIROCHAETES**			–	**2.4**	–	–	
	Leptospiraceae		–	2.4 (1)	–	–	
**NITROSPIRAEA**			**3.4**	–	**1.4**	–	
	Nitrospiraceae		3.4 (1)	–	1.4 (1)	–	
**CHLOROFLEXI**			–	**1.2**	–	–	
	Caldilinelaceae		–	–	–	–	
	Unclassified	–	–	–	–	–	
**CHLOROBI**			**1.1**	–	–	–	
	Unclassified		–	–	–	–	

The numbers of OTUs are in brackets.

Focusing on *Gammaproteobacteria* and *Deltaproteobacteria*, the most abundant OTUs, it is important to note that most of the sequences of the four T_270d_ libraries were associated with microorganisms involved in the sulfur cycle (Table 2, see Figs. S7 and S8 in the supplemental material). They included sulfur-oxidizing bacteria (SOB) such as purple sulfur bacteria within the *Chromatiaceae* and *Ectothiorhodospiraceae* families, sulfur-reducing *Desulforomonadaceae* and sulfate-reducing bacteria (*Desulfobacteraceae* and *Desulfobulbaceae*). The NEREIS library was characterized by 74.9% of sequences related to these families (about 40% in the other libraries). They were mainly associated with SOB (56.9%) as Sulfur-oxidizing bacterium ODIII6, *Thiohalophilus thiocyanatoxydans* and *Olavius algarvensis* sulfur-oxidizing endosymbiont. *Chromatiaceae* were found in lower abundance in the BAL and NEREIS+BAL libraries than in the CTRL and NEREIS libraries, indicating that they were affected by oil. In contrast, we observed that the sulfate-reducing *Desulfobacteraceae* were abundant (15.9% and 13.5%) in oiled libraries. The abundance of *Alteromonadacae* was higher in microcosms to which *H. diversicolor* had been added.

The phylogenetic analysis also revealed the presence of sequences associated with bacteria known to be able to degrade hydrocarbons (see Fig. S10 in the supplemental material). They were more abundant in oiled microcosms accounting for 14.5% (6 OTUs) and 18.8% (8 OTUs) of sequences in the BAL and NEREIS+BAL libraries respectively. The sequences in the BAL library were related to *Betaproteobacteria* and sulfate-reducing strains of *Desulfococcus* and *Desulfosarcina* genera while the NEREIS+BAL library was characterized by the presence of sequences related to members of *Gammaproteobacteria* and sulfate-reducing strains of *Desulfobacterium* genus, known for their ability to degrade hydrocarbons (Fig. S10).

## Discussion

In this study, we found that the structure and diversity of bacterial communities of marine sediment were affected by oil addition and the polychaete. Depending on the addition of the polychaetes *Hediste (Nereis) diversicolor* different bacterial communities were obtained without affecting the overall oil removal capacity. However, we did not find evidence to support the hypothesis that burrowing activity affected the bacterial community’s oil degradation capacity. We also did not observe any degradation of the heaviest PAHs, suggesting the absence of photo-oxidation. Without excluding other abiotic processes, we assume that microbial biodegradation of hydrocarbon compounds was effective due to the n-C18/phytane ratio. The NEREIS+BAL bacterial community showed higher diversity than the BAL bacterial community reflecting different structures with distinct sequences associated with bacteria known for their ability to degrade hydrocarbons. A few studies have reported that burrowing organisms, through mucus excretion and oxygenation of sediments, influence the bacterial communities in the burrow and its surroundings as well [35]–[37]. Their presence had an impact on the bacterial degradation capacities, stimulating microbial degradation of organic matter [38] and organic contaminants [39] including crude oil compounds [7].

In the present study, we also found evidence that *H. diversicolor*’s burrowing increased as previously reported [40] and that the burrowing was further enhanced in the presence of BAL 110 oil. Similar observations have been reported [5], but this was not the case during a laboratory experiment where *H. diversicolor* reworking was found to be reduced in the presence of Arabian Light crude oil [41]. In our study, the presence of oil would limit the penetration of oxygen, nutrients, and organic matter that are required for macrobenthic life [42], [43]. Thus, the observed enhancement of the reworking activity could be explained by the fact that polychaetes would limit their stay in deeper sediment zones. Regarding the effect of *H. diversicolor* and its reworking activity on the microbial degradation capacities contradictory observations were reported. It was shown that pyrene degradation was not increased by the presence of *H. diversicolor* [6] while another report showed that the mineralisation of naphthalene was decreased in the presence of high density meiofauna [44]. These studies, performed with single molecules, did not focus on the composition of active bacterial communities. Thus, to the best of our knowledge, this work represents the first attempt to study the impact of petroleum, a complex mixture, on the composition of active bacterial communities in bioturbated sediments.

T-RFLP (both DNA and cDNA analyses) showed that BAL and NEREIS+BAL bacterial community dynamics were oil-dependent. A first community shift was observed after 90 days of incubation in both contaminated BAL and NEREIS+BAL conditions followed by a second community shift after 180 days of incubation, suggesting that a succession of bacterial communities occurred, which resulted in different bacterial patterns according to the treatment, both showing oil removal capacities. This succession of bacterial communities could be related to the different phases of hydrocarbon removal observed, the earlier degradation of n-alkanes followed by degradation of PAHs. This is supported by other studies showing that bacterial communities are first dominated by n-alkane-degrading bacteria and then by bacterial groups degrading polyaromatic hydrocarbons (PAHs), compounds more difficult to degrade (for a review see [45]).

In the NEREIS+BAL condition, the number of OTUs detected only in the DNA but not in the RNA analyses (RNA− DNA+; representing inactive dominant fraction of the community) decreased suggesting that some OTUs became much more active to a point where they could be detected on the RNA-based analyses (RNA+ DNA+; representing the active dominant fraction of the community). The OTUs detected only in the RNA analyses (RNA+ DNA−; representing the active minor fraction of the community) were stable during the course of the experiment. The dominant fraction of the NEREIS+BAL community became active earlier than that of the BAL community, showing the role of polychaetes in the stimulation of the bacterial population. Furthermore, the significant correlation observed between the bacterial dominant active fractions and hydrocarbon concentrations may suggest the involvement of dominant active fraction of NEREIS+BAL and BAL communities in hydrocarbon biodegradation.

In oiled microcosms, the comparison of bacterial communities based on T-RFLP and 16S cDNA library analyses revealed that the bacterial communities showed different dynamics in microcosms to which the polychaetes was added, resulting in two distinct bacterial communities. The NEREIS+BAL active community was characterized by OTUs related to *Gamma-* and *Deltaproteobacteria* while the most active OTUs in the BAL community were related to *Alpha-* and *Deltaproteobacteria*. These results are consistent with recent Deepwater Horizon oil spill studies, showing that bacterial communities are predominated by members of the *Gamma-* and *Alphaproteobacteria* in oil-polluted sediments [46] or sand beach [47]. Indeed, sequences retrieved in our study are related to sequences of hydrocarbon-degrading isolates obtained from sediments contaminated by Deepwater Horizon oil spill, especially associated with members of *Cycloclasticus* and *Marinobacter* genera (see Fig. S10 in the supplemental material). It is also important to notice that we obtained sequences related to sequences retrieved in oil contaminated sediments, some of them related to sequences retrieved in Prestige oil contaminated sediments [48] (see Fig. S10 in the supplemental material). These observations indicated that hydrocarbon degraders were induced in our sediment. In our study, the phylogenetic affiliation of 16S cDNA sequences indicated that almost all sequences were related to bacterial groups involved in the sulfur cycle; only 14% of total phylotypes were associated with bacteria known to be able to degrade hydrocarbons. Many studies have reported the dominance of bacteria able to oxidize hydrocarbons in oil-contaminated ecosystems [3], [4], [46], [47], [49]–[51]. It is possible that our analysis missed the bacterial bloom related to alkane degraders occurring rapidly after oil contamination [45]. However, since the hydrocarbons were present until the end of the experiment (40% TPH at T_270d_), the low abundance of sequences associated with hydrocarbon-degrading-bacteria in our sediments could be explained by the presence of microorganisms capable of oxidizing hydrocarbons different from those already cultivated, as well as by the fact that the sediments were from a non-polluted environment. Only biogenic hydrocarbons were detected, harboring a bacterial community non-adapted to petroleum compounds. Several reports [52], [53] have demonstrated that the impact of crude oil on the bacterial community and the oil degradation capacities are dependent on the environment’s history of pollution events [52]. These studies show that oil degradation was faster when bacterial communities were adapted to oil compounds [53]. The slow degradation in our experimental sediments that were begun in the winter season, could be explained by their being maintained at low ambient temperatures. Indeed low temperatures affect the bio-availability of hydrocarbon compounds by increasing their viscosity and decreasing their volatility [54] as well as the microbial activities [55]. In the present study, sequences related to sulfur-oxidizing bacteria (SOB) were found in lower abundance in oiled libraries than in un-oiled libraries. Most of these sequences were related to the cultivable strains *Thioalkalivibrio denitrificans* and *Thiohalophilus thiocyanoxidans* [56], [57]. The ability of these strains to grow with different electron acceptors such as oxygen and nitrogen oxides has been shown, demonstrating their adaptation capacity to different conditions and their role in the sulfur, nitrogen, and carbon biogeochemical cycles 58.

Sulfate-reducing bacteria (SRB) were found to be dominant in oiled microcosms (BAL and NEREIS+BAL). The simultaneous presence of both SOB and SRB communities suggests that the sulfur cycle was active in the microcosms. SRB are known to play an important role in anaerobic oil degradation [59]–[61]. Previous studies have found SRBs to be dominant in oil contaminated sediments [62], [63], their activity being stimulated by the presence of hydrocarbons [64]. Interestingly in the present study, their abundance (based on library analyses) was not affected by the addition of *H. diversicolor*. This observation could be consistent with the increase in sulfate reduction rates observed in bioturbated sediments which other studies explained by the occurrence of reduced microniches through the increased burrowing activity [65]. However, other authors have also reported a decrease in sulfate-reduction rates in bioturbated sediment [12], [66]. Clearly, with the results in the present study the debate continues and more research is warranted.

Our results show that the bacterial community structure in mudflat sediments shows evidence of modification after petroleum addition. The modification was dependent on the presence of the added burrowing polychaetes *H. diversicolor* in the microcosms. Contrary to our initial hypothesis, the overall oil removal capacity was not affected by the addition of polychaetes, although it did show evidence of affecting bacterial community structure. Indeed, the addition of burrowing organisms stimulated the bioturbation activity, resulting in the selection of a particular bacterial community characterized by the dominance of *Gammaproteobacteria*, as previously described in shrimp-inhabited sediment [36]. In the presence of petroleum, the OTUs related to potential hydrocarbon-degrading bacteria were distinct from those observed in oiled microcosms without added *H. diversicolor*. Thus, there was a change in the bacterial community to two distinct bacterial communities, both showing a similar overall oil removal capacity. Several hypotheses explaining how environmental perturbations may affect bacterial community composition have been drawn; they can become more resistant, resilient, or functionally redundant [67], [68]. The mechanisms underlying functional redundancy are difficult to study because manipulating bacterial communities in the field is far from easy. In our study, the addition of burrowing organisms to sediments maintained near-environmental conditions allowed the manipulation of bacterial community structure and composition, opening the way for such a study. Furthermore, understanding the structuring effect of the reworking processes on bacterial communities will be useful for the management of bacterial metabolisms involved in bioremediation processes of coastal marine sediments.

## Supporting Information

Figure S1 **Distribution profiles of luminophores in the different conditions.** CTRL (white square), BAL (red square), NEREIS (white triangle) and NEREIS+BAL (red triangle).
(TIF)

Figure S2 **Total petroleum hydrocarbons (TPH) content on 0–8 cm depth during the 270 days of the experiment for the BAL (gray bar) and NEREIS+BAL (dotted bar) conditions.** Stars show significant difference obtained by Duncan test (p-value<0.05) compared to 2 days value.
(TIF)

Figure S3 **n-C_18_/phytane ratio for BAL (dotted line) and NEREIS+BAL (solid line) during the 270 days of experiment.**
(TIF)

Figure S4 **nMDS analyses showing the temporal dynamic of bacterial community structures based on 16S rRNA gene (A) and transcripts (B) for 270 days for the four conditions (CTRL, white circle; BAL, black circle; NEREIS, white triangle; NEREIS+BAL, black triangle).** Stress values are indicated for each analysis.
(TIF)

Figure S5 **nMDS analyses showing treatment effects (CTRL, BAL, NEREIS, NEREIS+BAL) on bacterial community structures based on 16S rRNA gene (A) and transcripts (B) for 270 days for the four conditions.** The ellipses indicate a 95% confidence interval for replicates. Stress values are indicated for each analysis.
(TIF)

Figure S6 **Distribution and abundance (%) of operational taxonomic units (OTUs) from T-RFLP fingerprinting of 16S rRNA genes and 16S rRNA transcripts obtained at 2 days, 180 days and 270 days of incubation for BAL (A) and NEREIS+BAL (B) conditions.** Squares represent individual OTU. Size of the squares is related to relative abundance (% of fluorescence intensity of each OTU). Gray, OTUs common to 16S rRNA genes and 16S rRNA transcripts profiles.
(TIF)

Figure S7 **Phylogenetic tree based on the analysis of 16S rRNA transcripts cloned sequences (844 bp aligned) from CTRL (white circle), BAL (black circle), NEREIS (white triangle) and NEREIS+BAL (black triangle) conditions at 270 days.** Only sequences and their closest relative sequences affiliated to *Gammaproteobacteria*are shown. The scale bar corresponds to 0.02 substitutions per nucleotide. Percentages of 1000 bootstrap resampling are shown above or near the relevant nodes. Bootstrap values are shown for branches with more than 50% bootstrap support. The relative abundance of clones in the corresponding microcosm library is notified. Sequences from this study are underlined.
(TIF)

Figure S8 **Phylogenetic tree based on the analysis of 16S rRNA transcripts cloned sequences (844 bp aligned) from CTRL (white circle), BAL (black circle), NEREIS (white triangle) and NEREIS+BAL (black triangle) conditions at 270 days.** Only clones and their closest relative sequences affiliated to *Proteobacteria,* others than *Gammaproteobacteria,* are shown. The scale bar corresponds to 0.02 substitutions per nucleotide. Percentages of 1000 bootstrap resampling are shown above or near the relevant nodes. Bootstrap values are shown for branches with more than 50% bootstrap support. The relative abundance of clones in the corresponding microcosm library is notified. Sequences from this study are underlined, sequences from cultivable strains are shown in bold and uncultured clones sequences are shown in gray.
(TIF)

Figure S9 **Phylogenetic tree based on the analysis of 16S rRNA transcripts cloned sequences (844 bp aligned) from CTRL (white circle), BAL (black circle), NEREIS (white triangle) and NEREIS+BAL (black triangle) conditions at 270 days.** Only clones and their closest relative sequences affiliated to others phyla than *Proteobacteria* are shown. The scale bar corresponds to 0.02 substitutions per nucleotide. Percentages of 1000 bootstrap resampling are shown above or near the relevant nodes. Bootstrap values are shown for branches with more than 50% bootstrap support. The relative abundance of clones in the corresponding microcosm library is notified. Sequences from this study are underlined, sequences from cultivable strains are shown in bold and uncultured clones sequences are shown in gray.
(TIF)

Figure S10 **Phylogenetic tree of sequences related to hydrocarbon degrading bacteria.** The analysis is based on sequences of 16S rRNA transcripts (844 bp aligned) found in the libraries of oil-polluted microcosms BAL (gray) and NEREIS+BAL (black) at 270 days. Sequence found in both libraries isin box. The scale bar corresponds to 0.02 substitutions per nucleotide. Percentages of 1000 bootstrap resampling are shown above or near the relevant nodes. Bootstrap values are shown for branches with more than 50% bootstrap support. Sequences from cultivable strains are shown in bold. Black dots correspond to sequences of oil-degrading bacteria. Sequences of hydrocarbon-degrading isolates from sediments contaminated by Deepwater Horizon oil spill are in red. Sequences obtained from Prestige oil contaminated sediments are in blue.
(TIF)

Table S1 **Average dissimilarity (estimated with a Bray-Curtis distance) among communities for each treatment.** (Mean ±SD; SD = standard deviation).
(DOCX)

Table S2 **Results of ANOVAs to test for a) the effects of NEREIS addition, time and depth on TPH content and b) the effects of NEREIS addition and time on the biodegradable fraction content (** ***n*** **-alkanes and PAHs).**
(DOCX)

Table S3 **Results of permutational multivariate analysis of variance (PerMANOVA) to test for the effects of time, BAL addition and NEREIS addition on relative abundance of DNA OTUS and cDNA OTUs.** In the model I, permutations were constrained within biological replicates (BRs) to take into account the repeated measures; model I allows proper estimations of the effects of time and its interactions with other factors. In the model II, permutations occurred within each sampling time in order to obtain proper estimations of the BAL and NEREIS effects.
(DOCX)

Table S4 **Bacterial community structure comparison based on T-RFLP analyses.** ANOSIM test values for analyses between the different bacterial community clusters (Figure 4A, 4B). p-value indicates the significance level.
(DOCX)
