# Supplementary figures and images for: Impact of Oil on Bacterial Community Structure in Bioturbated Sediments
Source: PLoS One. 2013 Jun 10;8(6):e65347. doi: 10.1371/journal.pone.0065347 (PMC3677869; doi:10.1371/journal.pone.0065347)

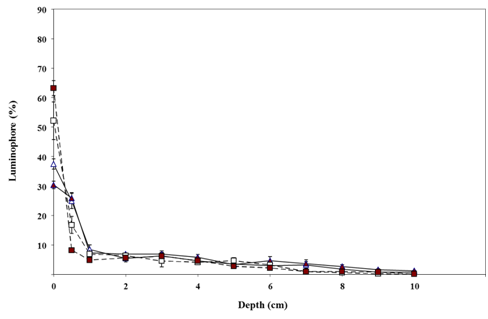

Supplement: Figure S1 — Distribution profiles of luminophores in the different conditions. CTRL (white square), BAL (red square), NEREIS (white triangle) and NEREIS+BAL (red triangle). (TIF) [file pone.0065347.s001.tif]

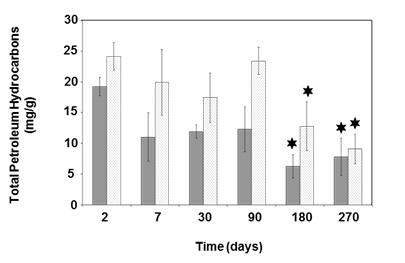

Supplement: Figure S2 — Total petroleum hydrocarbons (TPH) content on 0–8 cm depth during the 270 days of the experiment for the BAL (gray bar) and NEREIS+BAL (dotted bar) conditions. Stars show significant difference obtained by Duncan test (p-value<0.05) compared to 2 days value. (TIF) [file pone.0065347.s002.tif]

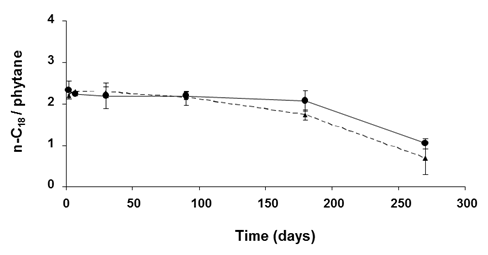

Supplement: Figure S3 — n-C18/phytane ratio for BAL (dotted line) and NEREIS+BAL (solid line) during the 270 days of experiment. (TIF) [file pone.0065347.s003.tif]

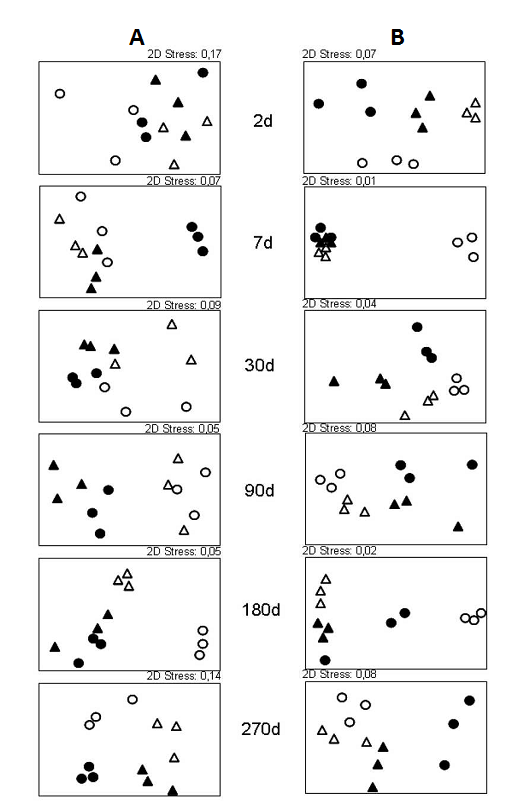

Supplement: Figure S4 — nMDS analyses showing the temporal dynamic of bacterial community structures based on 16S rRNA gene (A) and transcripts (B) for 270 days for the four conditions (CTRL, white circle; BAL, black circle; NEREIS, white triangle; NEREIS+BAL, black triangle). Stress values are indicated for each analysis. (TIF) [file pone.0065347.s004.tif]

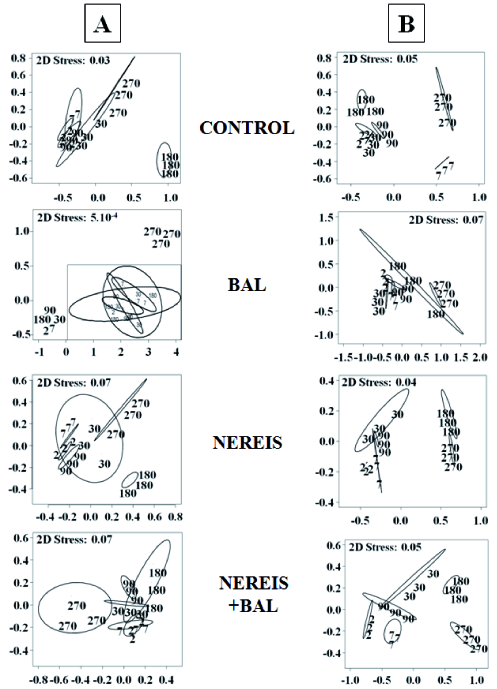

Supplement: Figure S5 — nMDS analyses showing treatment effects (CTRL, BAL, NEREIS, NEREIS+BAL) on bacterial community structures based on 16S rRNA gene (A) and transcripts (B) for 270 days for the four conditions. The ellipses indicate a 95% confidence interval for replicates. Stress values are indicated for each analysis. (TIF) [file pone.0065347.s005.tif]

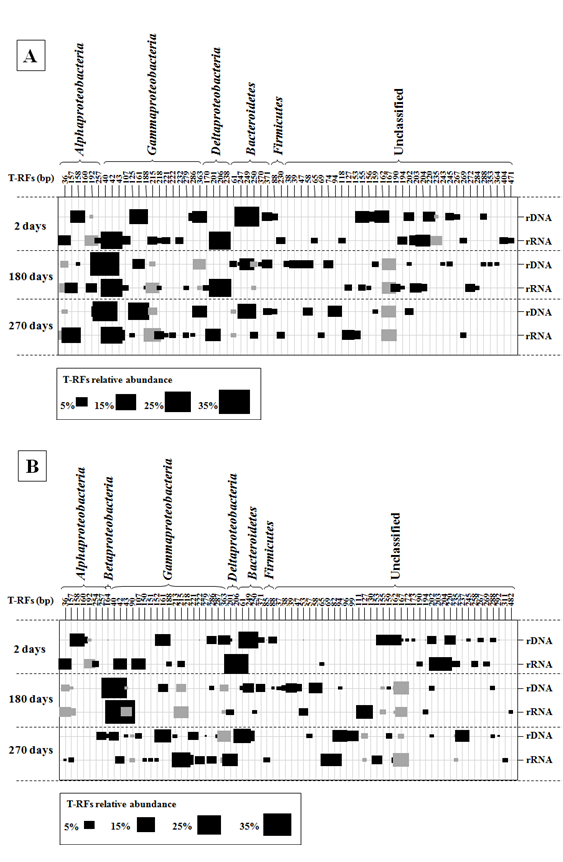

Supplement: Figure S6 — Distribution and abundance (%) of operational taxonomic units (OTUs) from T-RFLP fingerprinting of 16S rRNA genes and 16S rRNA transcripts obtained at 2 days, 180 days and 270 days of incubation for BAL (A) and NEREIS+BAL (B) conditions. Squares represent individual OTU. Size of the squares is related to relative abundance (% of fluorescence intensity of each OTU). Gray, OTUs common to 16S rRNA genes and 16S rRNA transcripts profiles. (TIF) [file pone.0065347.s006.tif]

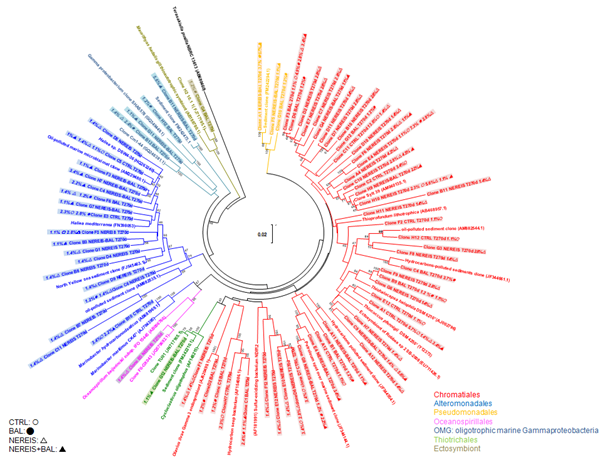

Supplement: Figure S7 — Phylogenetic tree based on the analysis of 16S rRNA transcripts cloned sequences (844 bp aligned) from CTRL (white circle), BAL (black circle), NEREIS (white triangle) and NEREIS+BAL (black triangle) conditions at 270 days. Only sequences and their closest relative sequences affiliated to Gammaproteobacteriaare shown. The scale bar corresponds to 0.02 substitutions per nucleotide. Percentages of 1000 bootstrap resampling are shown above or near the relevant nodes. Bootstrap values are shown for branches with more than 50% bootstrap support. The relative abundance of clones in the corresponding microcosm library is notified. Sequences from this study are underlined. (TIF) [file pone.0065347.s007.tif]

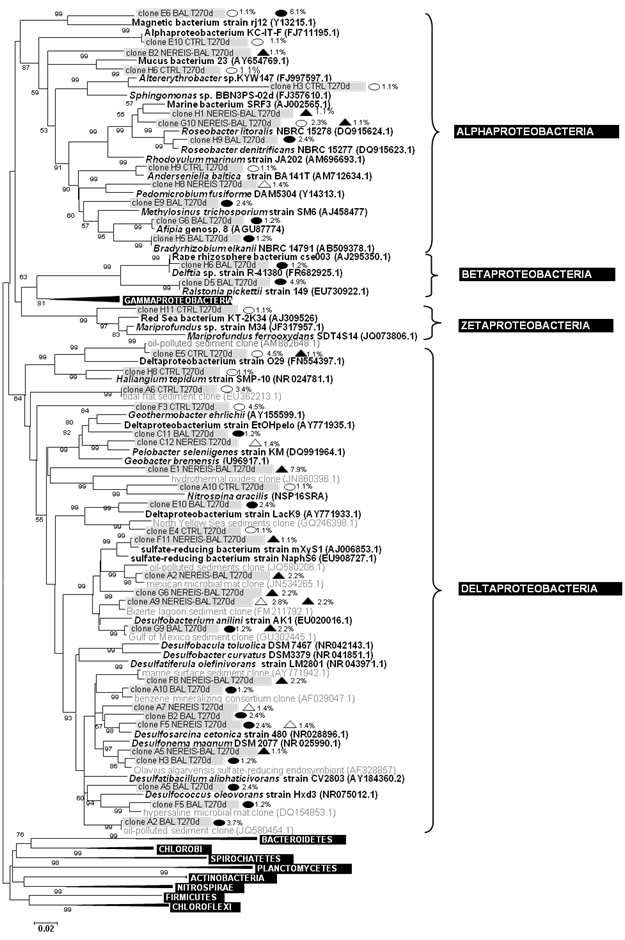

Supplement: Figure S8 — Phylogenetic tree based on the analysis of 16S rRNA transcripts cloned sequences (844 bp aligned) from CTRL (white circle), BAL (black circle), NEREIS (white triangle) and NEREIS+BAL (black triangle) conditions at 270 days. Only clones and their closest relative sequences affiliated to Proteobacteria, others than Gammaproteobacteria, are shown. The scale bar corresponds to 0.02 substitutions per nucleotide. Percentages of 1000 bootstrap resampling are shown above or near the relevant nodes. Bootstrap values are shown for branches with more than 50% bootstrap support. The relative abundance of clones in the corresponding microcosm library is notified. Sequences from this study are underlined, sequences from cultivable strains are shown in bold and uncultured clones sequences are shown in gray. (TIF) [file pone.0065347.s008.tif]

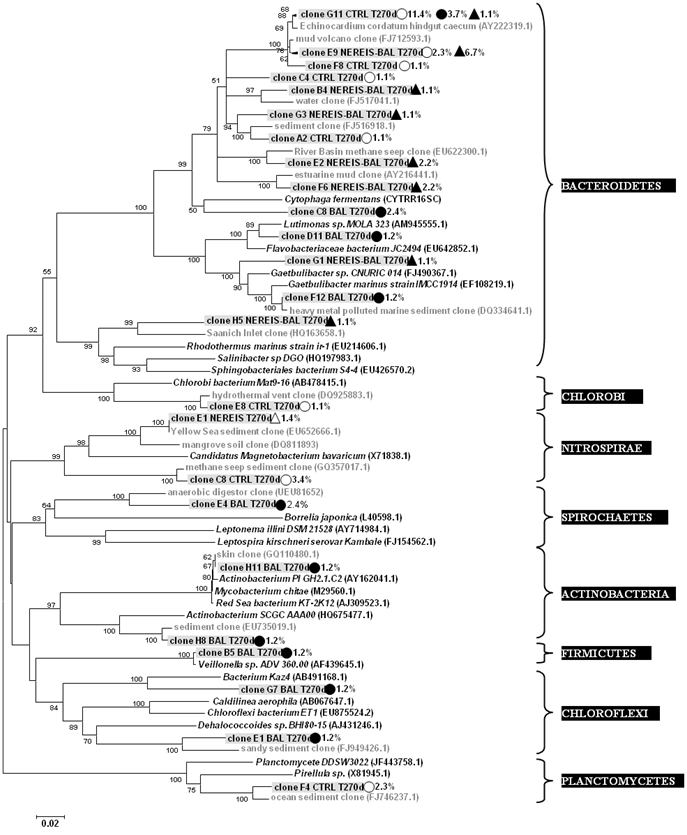

Supplement: Figure S9 — Phylogenetic tree based on the analysis of 16S rRNA transcripts cloned sequences (844 bp aligned) from CTRL (white circle), BAL (black circle), NEREIS (white triangle) and NEREIS+BAL (black triangle) conditions at 270 days. Only clones and their closest relative sequences affiliated to others phyla than Proteobacteria are shown. The scale bar corresponds to 0.02 substitutions per nucleotide. Percentages of 1000 bootstrap resampling are shown above or near the relevant nodes. Bootstrap values are shown for branches with more than 50% bootstrap support. The relative abundance of clones in the corresponding microcosm library is notified. Sequences from this study are underlined, sequences from cultivable strains are shown in bold and uncultured clones sequences are shown in gray. (TIF) [file pone.0065347.s009.tif]

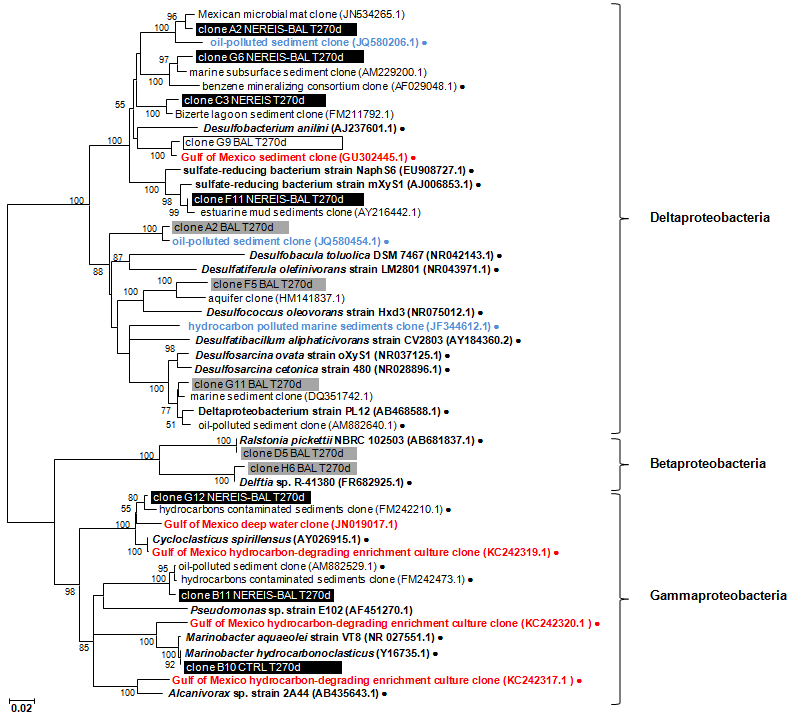

Supplement: Figure S10 — Phylogenetic tree of sequences related to hydrocarbon degrading bacteria. The analysis is based on sequences of 16S rRNA transcripts (844 bp aligned) found in the libraries of oil-polluted microcosms BAL (gray) and NEREIS+BAL (black) at 270 days. Sequence found in both libraries isin box. The scale bar corresponds to 0.02 substitutions per nucleotide. Percentages of 1000 bootstrap resampling are shown above or near the relevant nodes. Bootstrap values are shown for branches with more than 50% bootstrap support. Sequences from cultivable strains are shown in bold. Black dots correspond to sequences of oil-degrading bacteria. Sequences of hydrocarbon-degrading isolates from sediments contaminated by Deepwater Horizon oil spill are in red. Sequences obtained from Prestige oil contaminated sediments are in blue. (TIF) [file pone.0065347.s010.tif]
