# Supplementary material for: Impact of Oil on Bacterial Community Structure in Bioturbated Sediments
Source: PLoS One. 2013 Jun 10;8(6):e65347. doi: 10.1371/journal.pone.0065347 (PMC3677869; doi:10.1371/journal.pone.0065347)
Supplement: Table S1 — Average dissimilarity (estimated with a Bray-Curtis distance) among communities for each treatment. (Mean ±SD; SD = standard deviation). (DOCX) [file pone.0065347.s011.docx]

| **Treatment** | **Time (days)** | **Mean dissimilarity ±SD** |
| --- | --- | --- |
| CTRL | 2 | 0.25 ± 0.03 |
| CTRL | 7 | 0.36 ± 0.08 |
| CTRL | 30 | 0.45 ± 0.08 |
| CTRL | 90 | 0.22 ± 0.02 |
| CTRL | 180 | 0.28 ± 0.09 |
| CTRL | 270 | 0.44 ± 0.12 |
| BAL | 2 | 0.24 ± 0.06 |
| BAL | 7 | 0.14 ± 0.02 |
| BAL | 30 | 0.23 ± 0.03 |
| BAL | 90 | 0.18 ± 0.04 |
| BAL | 180 | 0.34 ± 0.07 |
| BAL | 270 | 0.23 ± 0.07 |
| NEREIS | 2 | 0.20 ± 0.04 |
| NEREIS | 7 | 0.16 ± 0.07 |
| NEREIS | 30 | 0.42 ± 0.02 |
| NEREIS | 90 | 0.25 ± 0.04 |
| NEREIS | 180 | 0.21 ± 0.04 |
| NEREIS | 270 | 0.42 ± 0.08 |
| BAL + NEREIS | 2 | 0.22 ± 0.02 |
| BAL + NEREIS | 7 | 0.24 ± 0.08 |
| BAL + NEREIS | 30 | 0.26 ± 0.06 |
| BAL + NEREIS | 90 | 0.18 ± 0.02 |
| BAL + NEREIS | 180 | 0.35 ± 0.12 |
| BAL + NEREIS | 270 | 0.37 ± 0.07 |
