# Supplementary material for: Impact of Oil on Bacterial Community Structure in Bioturbated Sediments
Source: PLoS One. 2013 Jun 10;8(6):e65347. doi: 10.1371/journal.pone.0065347 (PMC3677869; doi:10.1371/journal.pone.0065347)
Supplement: Table S2 — Results of ANOVAs to test for a) the effects of NEREIS addition, time and depth on TPH content and b) the effects of NEREIS addition and time on the biodegradable fraction content ( n -alkanes and PAHs). (DOCX) [file pone.0065347.s012.docx]

|  |  | **Df** | **SS^1^** | **MS^2^** | **F-statistic** | **p-value** |
| --- | --- | --- | --- | --- | --- | --- |
| **a) TPH content** | |  |  |  |  |  |
|  | NEREIS (N) | 2 | 364.86 | 182.43 | 682.08 | <0.0001 |
|  | Time (T) | 5 | 12.27 | 2.45 | 9.18 | <0.0001 |
|  | Depth (D) | 3 | 212.21 | 70.74 | 264.47 | <0.0001 |
|  | N : T | 5 | 4.65 | 0.93 | 3.48 | 0.0062 |
|  | N : D | 3 | 6.13 | 2.04 | 7.64 | 0.00012 |
|  | D : T | 15 | 21.14 | 1.41 | 5.27 | <0.0001 |
|  | N : P : T | 15 | 4.68 | 0.31 | 1.17 | 0.31 |
|  | Residuals | 96 | 25.68 | 0.27 |  |  |
| **b) *n*-alkane content** | |  |  |  |  |  |
|  | NEREIS (N) | 1 | 1.45 | 1.44 | 5.94 | 0.03 |
|  | Time (T) | 5 | 16.27 | 3.25 | 13.36 | <0.0001 |
|  | N : T | 5 | 0.78 | 0.15 | 0.64 | 0.67 |
|  | Residuals | 23 | 5.6 | 0.24 |  |  |
| **PAH content** | |  |  |  |  |  |
|  | NEREIS (N) | 1 | 1.57 | 1.57 | 9.33 | 0.0056 |
|  | Time (T) | 5 | 3.68 | 0.74 | 4.36 | 0.0061 |
|  | N : T | 5 | 0.54 | 0.11 | 0.64 | 0.68 |
|  | Residuals | 23 | 3.885 | 0.17 |  |  |

SS^1^: Sum of square;MS^2^: Mean of squares
