# Supplementary material for: Impact of Oil on Bacterial Community Structure in Bioturbated Sediments
Source: PLoS One. 2013 Jun 10;8(6):e65347. doi: 10.1371/journal.pone.0065347 (PMC3677869; doi:10.1371/journal.pone.0065347)
Supplement: Table S3 — Results of permutational multivariate analysis of variance (PerMANOVA) to test for the effects of time, BAL addition and NEREIS addition on relative abundance of DNA OTUS and cDNA OTUs. In the model I, permutations were constrained within biological replicates (BRs) to take into account the repeated measures; model I allows proper estimations of the effects of time and its interactions with other factors. In the model II, permutations occurred within each sampling time in order to obtain proper estimations of the BAL and NEREIS effects. (DOCX) [file pone.0065347.s013.docx]

|  | **DNA OTUs** | | | | | **cDNA OTUs** | | | | |
| --- | --- | --- | --- | --- | --- | --- | --- | --- | --- | --- |
|  | **Df** | **SS^1^** | **MS^2^** | **F-statistic** | **p-value** | **Df** | **SS^1^** | **MS^2^** | **F-statistic** | **p-value** |
| **Model I** |  |  |  |  |  |  |  |  |  |  |
| *Time (T)* | 5 | 6.7 | 1.34 | 29.63 | 0.001 | 5 | 7.65 | 1.53 | 33.49 | 0.001 |
| *BAL effect (B)* | 1 | 0.75 | 0.75 | 16.63 | 0.001 | 1 | 0.54 | 0.54 | 11.78 | 0.001 |
| *NEREIS effect (N)* | 1 | 0.24 | 0.24 | 5.24 | 0.001 | 1 | 0.45 | 0.45 | 9.81 | 0.001 |
| *Microcosm boxes* | 9 | 0.44 | 0.05 | 1.09 | 0.001 | 9 | 0.48 | 0.05 | 1.17 | 0.001 |
| *T : B* | 5 | 1.37 | 0.27 | 6.08 | 0.001 | 5 | 1.71 | 0.34 | 7.48 | 0.001 |
| *T : N* | 5 | 1.17 | 0.23 | 5.15 | 0.001 | 5 | 2.24 | 0.45 | 9.79 | 0.001 |
| *T : B : N* | 5 | 0.77 | 0.15 | 3.4 | 0.001 | 5 | 1.12 | 0.22 | 4.89 | 0.001 |
| *residuals* | 40 | 1.81 | 0.05 |  |  | 40 | 1.83 | 0.05 |  |  |
| *Total* | 71 | 13.26 |  |  |  | 71 | 16.02 |  |  |  |
| **Model II** |  |  |  |  |  |  |  |  |  |  |
| *Time (T)* | 5 | 6.7 | 1.34 | 15.56 | 0.001 | 5 | 7.65 | 1.53 | 13.42 | 0.001 |
| *BAL effect (B)* | 1 | 0.75 | 0.75 | 8.74 | 0.001 | 1 | 0.54 | 0.54 | 4.72 | 0.001 |
| *NEREIS effect (N)* | 1 | 0.24 | 0.24 | 2.75 | 0.007 | 1 | 0.45 | 0.45 | 3.93 | 0.004 |
| *B : N* | 1 | 0.14 | 0.14 | 1.68 | 0.091 | 1 | 0.19 | 0.19 | 1.68 | 0.114 |
| *residuals* | 63 | 5.43 | 0.09 |  |  | 63 | 7.19 | 0.11 |  |  |
| *Total* | 71 | 13.26 |  |  |  | 71 | 16.02 |  |  |  |

SS^1^: Sum of square;MS^2^: Mean of squares
