# Supplementary material for: Impact of Oil on Bacterial Community Structure in Bioturbated Sediments
Source: PLoS One. 2013 Jun 10;8(6):e65347. doi: 10.1371/journal.pone.0065347 (PMC3677869; doi:10.1371/journal.pone.0065347)
Supplement: Table S4 — Bacterial community structure comparison based on T-RFLP analyses. ANOSIM test values for analyses between the different bacterial community clusters (Figure 4A, 4B). p-value indicates the significance level. (DOCX) [file pone.0065347.s014.docx]

|  | | **R-statistic** | **p-value** |
| --- | --- | --- | --- |
| **Total bacterialcommunity (DNA)** | |  |  |
| *Overall test* |  | 0.248 | 0.001 |
| *Pairwise comparisons of clusters* | | |  |
| I vs II |  | 0.747 | 0.001 |
| I vs III |  | 0.962 | 0.001 |
| II vs III |  | 0.782 | 0.001 |
| **Active bacterialcommunity (cDNA)** | |  |  |
| *Overall test* |  | 0.076 | 0.08 |
| *Pairwise comparisons of clusters* | | |  |
| IV vs V |  | 0.703 | 0.001 |
| IV vs VI |  | 0.776 | 0.001 |
| IV vs VII |  | 1 | 0.001 |
| V vs VI |  | 0.722 | 0.002 |
| V vs VII |  | 1 | 0.002 |
| VI vs VII |  | 1 | 0.002 |
